# T Cell Trafficking through Lymphatic Vessels

**DOI:** 10.3389/fimmu.2016.00613

**Published:** 2016-12-21

**Authors:** Morgan C. Hunter, Alvaro Teijeira, Cornelia Halin

**Affiliations:** ^1^Institute of Pharmaceutical Sciences, ETH Zurich, Zurich, Switzerland; ^2^Immunology and Immunotherapy Department, CIMA, Universidad de Navarra, Pamplona, Spain

**Keywords:** T cells, migration, trafficking, afferent, efferent, lymphatic vessels, lymph node

## Abstract

T cell migration within and between peripheral tissues and secondary lymphoid organs is essential for proper functioning of adaptive immunity. While active T cell migration within a tissue is fairly slow, blood vessels and lymphatic vessels (LVs) serve as speedy highways that enable T cells to travel rapidly over long distances. The molecular and cellular mechanisms of T cell migration out of blood vessels have been intensively studied over the past 30 years. By contrast, less is known about T cell trafficking through the lymphatic vasculature. This migratory process occurs in one manner within lymph nodes (LNs), where recirculating T cells continuously exit into efferent lymphatics to return to the blood circulation. In another manner, T cell trafficking through lymphatics also occurs in peripheral tissues, where T cells exit the tissue by means of afferent lymphatics, to migrate to draining LNs and back into blood. In this review, we highlight how the anatomy of the lymphatic vasculature supports T cell trafficking and review current knowledge regarding the molecular and cellular requirements of T cell migration through LVs. Finally, we summarize and discuss recent insights regarding the presumed relevance of T cell trafficking through afferent lymphatics.

## Introduction

In an antigen-inexperienced host, the frequency of naïve T cells specific for any given antigen is extremely low, several thousand at most (1, 2). Given that the diversity of possible antigens is almost countless and that T cell activation requires direct contact with antigen, naïve T cells constantly circulate through secondary lymphoid organs (SLOs) in pursuit of antigen (1, 2). Upon encountering antigen in SLOs, antigen-specific naïve T cells proliferate and become activated effector T cells (T_eff_) that egress from SLOs and enter peripheral tissue at sites of inflammation (2, 3). Most T_eff_ die after antigen is cleared but a few antigen-experienced T cells remain for long-term protection and either develop into tissue-resident memory T cells (T_RM_), into central memory T cells (T_CM_) that recirculate between SLOs and blood, or into effector-memory T cells (T_EM_) that circulate through blood and home to inflamed tissue (1, 2). In addition to the abovementioned antigen-experienced cell types, regulatory T cells (T_regs_) also circulate between blood, tissue, and SLOs (2–4).

Throughout the life of a T cell, the blood and lymphatic vasculature act as highways for T cell circulation. While much is known about T cell migration across and within the blood vasculature, much less is known about T cell migration into and within the lymphatic vasculature. Since the late 1950s, cannulation studies in sheep and rats have helped develop our current understanding of the cell subsets that circulate through lymphatic vessels (LVs). More recent technical advances (summarized in Box 1) have helped to further improve our understanding of the cellular and molecular mechanisms of T cell migration through LVs. In this review, we first introduce the structure of the lymphatic vascular system and summarize current knowledge of the cellular composition of efferent and afferent lymph. We then review the mechanisms by which T cells exit from lymph nodes (LNs) into efferent lymphatics as well as emerging knowledge of T cell entry and migration within afferent lymphatics. Finally, new insights regarding the overall relevance of T cell circulation through the afferent lymphatic vasculature are discussed.

Box 1 **Tools to study T cell trafficking *in vivo*.**

**Table:** 

Tool	Description	Selected reference
Cannulation studies	This procedure involves the surgical insertion of cannula (tube) directly into an afferent or efferent vessel or into the cisterna chyli, to collect lymph fluid. The cellular composition of lymph is subsequently analyzed, typically by flow cytometry or microscopy methods	(5–9)

Adoptive transfer	In adoptive transfer experiments, cells are isolated from donor mice, fluorescently labeled (unless already marked by endogenous expression of a fluorophore or a congenic marker) and intravenously or subcutaneously injected into a recipient mouse. In some cases, T cells are subjected to an *in vitro* culturing step (e.g., *in vitro* activation) prior to injection. At defined time points after transfer, T cell numbers in lymph nodes (LNs) (or other tissue) are quantified by flow cytometry, LN sectioning and microscopy, or other means. While this experimental setup is technically straightforward, the transferred cells may differ from the endogenously migrating populations. Also, typically only a small fraction of cells injected subcutaneously actually migrate to dLNs or beyond	(7, 10–13)

Intravital microscopy (IVM)	This technique allows the study of migratory processes at the single-cell level and in real time. It involves fluorescence-based time-lapse imaging by, e.g., confocal-/multiphoton- or stereomicroscopy. Several mouse reporter lines expressing a fluorescent protein in lymphatic vessels (LVs) have been generated (14–18). In the case of T cells, most studies have been performed with fluorescently labeled and adoptively transferred T cells, but endogenous models are also available (19–21)	(22–26)

Intralymphatic injection	Microinjection of T cells directly into a LV upstream of a draining lymph node. Similar to adoptive transfer but permits the study of T cell entry specifically across the LN subcapsular sinus. This represents an elegant yet technically challenging method complementing IVM studies	(25)

LN egress studies	This experimental setup allows quantifying dwell time of T cells in LNs. In a typical experiment, fluorescently labeled T cells are first transferred intravenously into a recipient mouse. After an equilibration phase, further T cell ingress into LNs is blocked by administration of entry-blocking antibodies (e.g., directed against the integrin subunit α4 or against L-selectin). Antibody treatment allows the uncoupling of T cell entry from exit, which continues to occur. Exit rates, for example, can be calculated by comparing fluorescent T cell numbers in LNs at the time of antibody injection to a later time point (e.g., 24 h later; flow cytometry-based quantification)	(9, 22, 27, 26)

Photoconvertible transgenic mice	The use of photoconvertible transgenic mice permits monitoring the migration of endogenously labeled cells *in vivo*. It requires transgenic mice expressing a photoconvertible fluorescent protein in all cell types [e.g., Kaede protein (28) or Kikume Green–Red protein (29)]. Upon illumination with violet light, fluorescent proteins undergo irreversible changes that alter their fluorescent spectrum (typically a green to red shift). By selectively illuminating the tissue at a particular site (e.g., skin), one can subsequently quantify the appearance of photoconverted T cells in other tissues (e.g., dLNs) to gain insight about their trafficking behavior. The system can easily be combined with pharmacologic blockade of genes of interest. Alternatively, backcrossing onto a genetic knockout can be done	(28, 30, 31)

## Structure of the Lymphatic Vasculature

**Figure 1:**
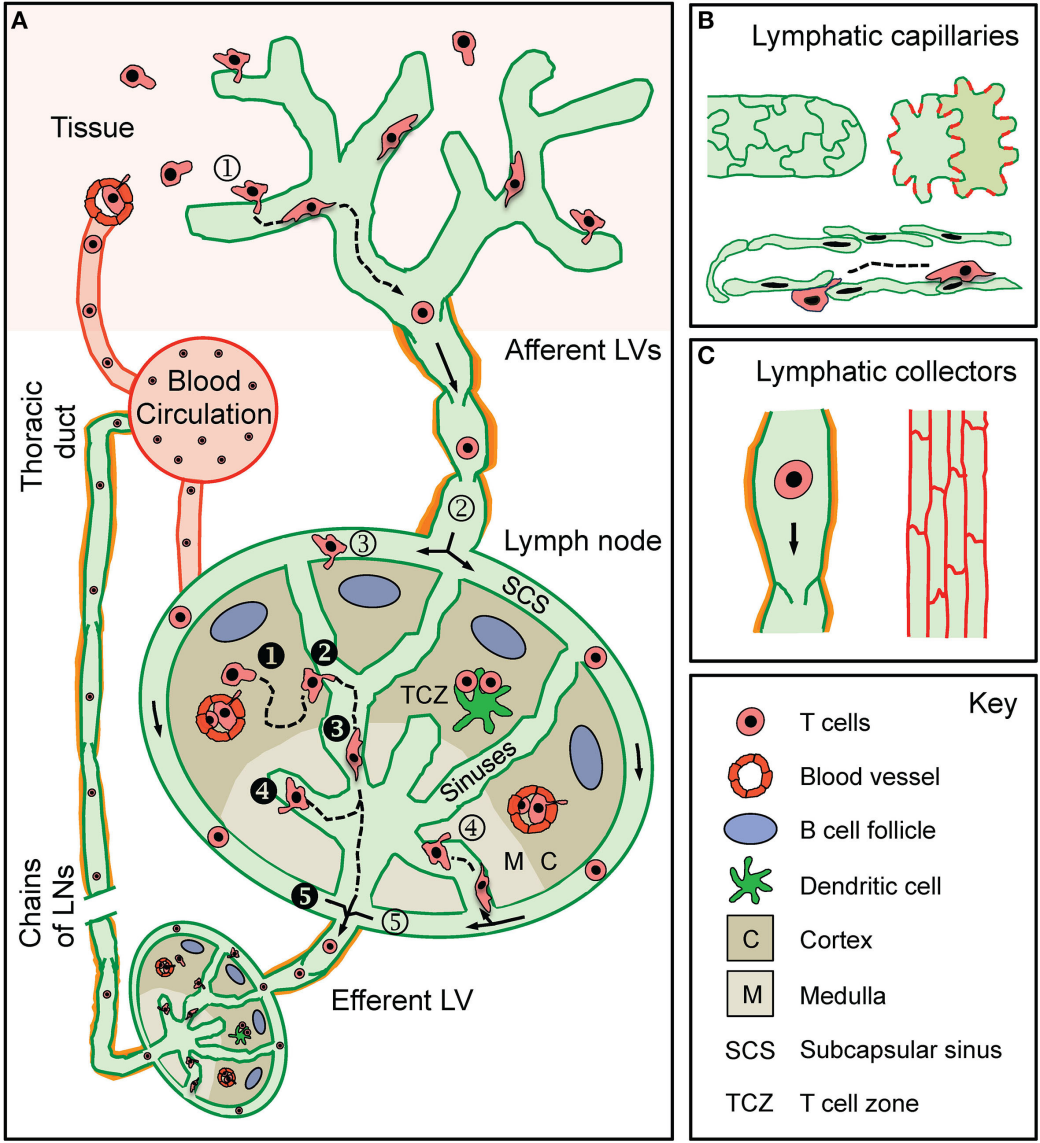
**T cell traffic through the lymphatic vascular system**. **(A)** Recirculating effector-memory T cells in peripheral tissues ➀ enter afferent lymphatic vessels (LVs). The exact point of entry or the mode of intralymphatic movement has not been investigated so far. T cells that ➁ arrive in the lymph node (LN) subcapsular sinus (SCS) have been shown to cross the lymphatic endothelium into the LN parenchyma at the level of the ➂ SCS or of the ➃ medullary sinuses. Some T cells do not enter the LN parenchyma but ➄ directly exit through the efferent LV located at the hilus region of the LN. Recirculating naïve and central memory T cells arrive in the LN either *via* the blood (high endothelial venules) or *via* the afferent LV draining from an upstream LN (i.e., efferent lymph). ❶ T cells within the LN ❷ make random contact with the sinuses before entering and ❸ actively crawling or passively flowing within the sinuses. T cells were observed to ❹ cross the sinuses several times before finally being ❺ passively carried away into the efferent LV. T cells in the efferent LV circulate through downstream LNs before being returned to the blood circulation via the thoracic duct. **(B)** Lymphatic capillaries are composed of oak leaf-shaped lymphatic endothelial cells (LECs), which partially overlap and are held together by button-like associated junctional adhesion molecules (red lines). This setup creates open flaps through which leukocytes, fluid, and macromolecules enter into the vessel lumen. **(C)** LECs in collecting vessels have a cuboidal shape and are connected by continuous cell-cell junctions (red lines). Collecting vessels contain intraluminal valves and are surrounded by a basement membrane and contracting smooth muscles cells (orange).

The lymphatic system consists of central and peripheral lymphoid organs and a LV network that permeates most tissues of the body (32, 33). In peripheral tissues, extravasated fluid, macromolecules, and leukocytes, i.e., the main constituents of lymph, are taken up by a network of blind-ended lymphatic capillaries, which converge into larger collecting vessels that drain into and through LNs (33). Upon passage through chains of tissue-draining LNs (dLNs), connected by adjoining collecting LVs, lymph is finally returned to the blood vasculature through the thoracic ducts, which merge into the subclavian vein (33) (Figure 1A).

Tissue fluid uptake and immune cell entry/transport into LVs is thought to mainly occur at the level of the initial lymphatic capillaries, where characteristic structural features support these processes. Lymphatic capillaries are composed of partially overlapping, oak leaf-shaped lymphatic endothelial cells (LECs) that are connected by discontinuous button-like cell–cell junctions (Figure 1B). Moreover, lymphatic capillaries are surrounded by a thin, highly fenestrated basement membrane (34, 35). Tissue fluid and leukocytes [as best shown for dendritic cells (DCs)] enter through the characteristic flaps between overlapping LECs (34, 35). Collecting LVs are structurally more specialized for fluid and immune cell transport (Figure 1C). Lymphatic collectors are composed of cuboidal LECs connected by continuous zipper-like cell–cell junctions and are surrounded by a continuous basement membrane and smooth muscle cell layer (34, 35). Intraluminal valves prevent the backflow of lymph, while contraction of smooth muscle cells helps to propagate lymph toward the dLN (36). Collecting vessels enter the LN and convey lymph along the subcapsular sinus (SCS) and through the LN sinuses toward the efferent LV in the hilus region (37) (Figure 1A). Efferent lymph is then transported in the efferent collecting vessel to downstream LNs and is finally returned to the blood vasculature. Considering that LNs in mice and humans are typically arranged in chains (38), the efferent LV of a tissue-draining LN is conjointly the afferent LV of the next downstream LN. In this review, we will consider afferent lymph as lymph that has not previously passed through a LN, i.e., lymph that is derived solely from non-lymphoid tissue (as designated in Figure 1A).

## Cellular Composition of Lymph

Most of our current knowledge on the cellular composition of lymph extends from cannulation studies (see Box 1). This relatively simple surgical model allows collection of lymph under physiologic conditions from a defined area of drainage over long periods of time (6, 39, 40)—and therefore most accurately reflects the composition of cells circulating through LVs. In rodents, efferent lymph can be collected from the cysterna chili in mice (8, 9, 41), or by cannulation of the thoracic duct in rats (5, 42). However, due to the small size of afferent LVs in mice and rats, cannulation of afferent LVs in rodents is very difficult. Correspondingly, most experimental studies comparing the composition of efferent and afferent lymph have been performed in larger animals like sheep (6, 7, 39, 40, 43–45).

### Efferent Lymph

Cannulation studies have revealed that thoracic duct lymph (46–48) as well as efferent lymph collected after passage through one or more LNs is mainly constituted by T lymphocytes (6, 43, 44). More than 90% of lymphocytes in efferent lymph were shown to have initially entered the LN through high endothelial venules (HEVs) (39, 43). CD4^+^ T cells enter and recirculate through LNs more rapidly than CD8^+^ T cells (27). Accordingly, CD4^+^ T cells constitute the major cellular fraction in efferent lymph and outnumber CD8^+^ T cells at a ratio higher than that in blood (49, 50). Most T cells in efferent lymph collected from sheep exhibit a naïve phenotype, with a reported increase in the proportion of memory T cells in older animals (44, 51, 52).

Antigenic stimulation of LNs often leads to distinct phases in the efferent lymph response: an initial “LN shutdown” where lymphocyte output is decreased; a “recruitment phase” where lymphocyte output rises above resting levels; and a “resolution phase” where lymphocyte output and cellular composition return to resting levels (53–55). While in most cases a sequential egress of CD4^+^ and then CD8^+^ T cells has been reported (56–58), the dominance of a particular lymphocyte subset in efferent lymph appears to be dependent on the antigenic stimulus (45, 59–61).

### Afferent Lymph

Compared to efferent lymph, the cellularity of afferent lymph is much lower (5–10%) under homeostatic conditions (6, 43, 44). While αβ T lymphocytes represent the most abundant cell type of afferent lymph (80-90%), DCs (5–15%), monocytes, B cells, and few granulocytes are also routinely found in steady-state afferent lymph (39, 43). CD4^+^ T cells in afferent lymph collected from sheep outnumber CD8^+^ T cells by approximately fourfold to fivefold (6, 43, 44). As reported in sheep, CD4^+^ T cells are the dominant cell type in afferent lymph collected from superficial dermal LVs of healthy humans (62–64). T cells in afferent lymph of both humans and sheep exhibit an effector-memory (T_EM_) phenotype, characterized by elevated expression of common T cell activation markers, adhesion molecules, and effector cytokines (44, 45, 63, 64). Although γδ T cells are present in large numbers in afferent lymph from sheep (65), they are almost non-existent in lymph or blood in humans (63, 64) and so are not further discussed here.

As cannulation of LVs is difficult in mice, a lot of our current knowledge of the T cell populations migrating through afferent LVs in mice has come from other experimental techniques used to investigate leukocyte trafficking (see Box 1). Specifically, these include adoptive transfer experiments or experiments performed in transgenic mice in which migrating leukocytes can be tracked by photoconversion of endogenously expressed fluorescent proteins [e.g., Kaede mice (28)—see Box 1]. Conclusions drawn from these approaches in mice are in accordance with earlier cannulation studies in larger animals. Moreover, they have revealed that the CD4^+^ T cell dominance in afferent lymph results from more efficient CD4^+^ T cell migration from the skin to the dLN (7, 31). In Kaede mice, the majority of CD4^+^ T cells that migrated from the skin to the dLN expressed the common T cell activation marker CD44 as well as the skin-homing molecules C–C chemokine receptor type 4 (CCR4) and E-selectin ligands (30, 31). Approximately 25% of CD4^+^ T cells that migrated from the skin to the dLN were also found to express the T_reg_ transcription factor FOXP3^+^ (30). Similarly, others have reported that adoptively transferred T_regs_ enter afferent LVs and migrate from the skin to dLN in mice (66–68). Notably, T_regs_ are phenotypically similar to T_EM_ and are only distinguishable when specific T_reg_ markers are used. The fact that FOXP3, the most widely used T_reg_ marker, was only described approximately 13 years ago might explain why T_regs_ have thus far not been reported from cannulation studies performed in sheep and humans (which frequently date back to earlier times).

In contrast to the conventional viewpoint that naïve T cells exclusively recirculate between blood and SLOs, low numbers of naïve T cells have also been found in both homeostatic and inflamed non-lymphoid tissues and have been suggested to circulate through afferent LVs (20, 69, 70). Indeed, in adoptive transfer experiments in mice, naïve T cells were shown to avidly migrate from the skin to dLN (7, 10). However, it is important to consider that the majority of endogenous CD4^+^ T cells in the skin have an effector/memory-like phenotype (10, 71). Correspondingly, cannulation studies in humans and sheep, and studies in Kaede mice, suggest that naïve T cells constitute only a minor subset of T cells in afferent lymph under both steady-state (30, 44, 64) and inflammatory conditions (12, 30, 72).

### Impact of Tissue Inflammation on Afferent Lymph Composition

Cannulation studies in sheep have revealed that acute skin inflammation, e.g., elicited by injection of complete Freund’s adjuvant (CFA), induced a dramatic increase in granulocyte numbers in skin-draining afferent lymph, whereas CD4^+^ and CD8^+^ T cells initially remained fairly stable (12, 53, 72, 73). By contrast, chronic inflammation, resulting from CFA-induced granuloma formation, was shown to lead to a substantial increase in CD4^+^ and CD8^+^ T cell output in skin-draining afferent lymph (12, 72). Contrastingly, in Kaede mice, an acute contact hypersensitivity response elicited a striking increase in the number of T cells that migrated from the skin to the dLN (30). However, it needs to be considered that numbers of T cells in steady-state lymph of laboratory mice might be unnaturally low, because of the sterile housing conditions that lead to the formation of a reduced pool of effector-memory T cells populating peripheral tissues (74).

## Recirculation of T Cells through Efferent Lymphatics

Seminal studies performed in the late 1950s by Sir James Gowans were the first to show that lymphocytes constantly circulate between blood and SLOs (42, 46). Naïve T cells in the blood extravasate through HEVs into the LN *via* a multistep adhesion cascade and subsequently migrate to T cell areas in the paracortex (75). Following entry into the LN, intranodal position, migration, and motility of T cells are mediated by C–C chemokine receptor type 7 (CCR7) and its two chemokine ligands, CCL19 and CCL21 (75, 76). Naïve T cells spend approximately 6–12 h surveying a LN for specific antigen and if undetected, transmigrate into cortical or medullary sinuses and exit through the efferent LV (28, 75). Below and in Table 1, we briefly review the chemotactic cues, adhesion molecules, and cellular processes involved in T cell egress from the LN into the efferent LVs.

**Table 1:** **Molecules regulating T cell exit from lymph nodes (LNs) through efferent lymphatic vessels (LVs)**.

Molecule	Selected reference	Comment
S1P_1_/S1P	(8, 9, 41, 77)	S1P_1_-deficient T cells are retained in LNs; disruption of S1P gradient in LNs prevents T cell egress
CD69	(78, 79)	CD69 expression induces S1P_1_ internalization and degradation in T cells resulting in T cell retention in LNs
C–C chemokine receptor type 7 (CCR7)	(22)	CCR7^−/−^ T cells egress more rapidly from LNs whereas CCR7 overexpressing T cells are retained
CXCR4	(80)	Synergizes with CCR7 in retaining T cells in LNs
Leukocyte function-associated antigen 1 (LFA-1)/intercellular adhesion molecule 1 (ICAM-1)	(26)	CD4^+^ LFA-1^−/−^ T cells egress more rapidly from LNs.
Common lymphatic endothelial and vascular endothelial receptor-1 (CLEVER-1)	(81)	Blockade of CLEVER-1 reduces T cell binding to LN sinuses *in situ*
		*In vivo* involvement not confirmed thus far
Mannose receptor (MR)/L-selectin	(82)	Blockade of MR/L-selectin reduces T cell binding to LN sinuses *in situ*
		*In vivo* involvement not demonstrated thus far
α9 integrin	(83)	Blockade of LEC-expressed α9 reduces T cell egress from LNs

### T Cell Egress vs. Retention: Interplay of S1P_1_, CD69, and CCR7

Early findings that pertussis toxin (a natural inhibitor of Gα_i_-protein-coupled receptors, such as chemokine receptors) inhibited the export of mature T cells from the thymus (84), suggested that egress of T cells from the LN could also be an active process. Studies on the immunosuppressive activity of Fingolimod (FTY720), a now approved treatment for multiple sclerosis (85), incited further research on the molecular mechanism of T cell exit from LNs. FTY720 induces sequestration of lymphocytes in SLOs through retention and “log jamming” of lymphocytes on the abluminal side of the lymphatic sinuses, thereby inhibiting lymphocyte egress into circulation and migration to sites of disease (86–88). Besides histologic analysis of lymphatic sinuses, efferent lymph cannulation studies and LN egress experiments, in which T cell homing into LNs is first blocked and T cell numbers subsequently quantified over time, have been instrumental for studying T cell exit into efferent LVs (see Box 1).

### Role of S1P

Several studies have shown that the egress-blocking activity of FTY720 can mainly be attributed to the action of FTY720 on sphingosine-1-phosphate (S1P) receptors, in particular, S1P receptor 1 (S1P_1_) expressed on T cells (8, 9, 89, 90). The natural ligand of S1P_1_ is S1P, an endogenous sphingolipid that mediates diverse cellular processes, including cell survival, cytoskeletal rearrangements, and cellular chemotaxis (91, 92). S1P levels in tissues are tightly controlled by sphingosine kinase 1 and 2 (Sphk1/2)-mediated production and S1P degradation, which depends on S1P lyase and other enzymes (77, 93). While erythrocytes, red blood cells, and the blood endothelium constitute major cellular sources of plasma S1P, lymph S1P is derived independently from the blood (91, 94). In fact, LECs were identified as the major source of S1P in lymph (41).

S1P levels in the blood and in lymph are much higher than in lymphoid organs (77, 95). Low concentrations of S1P in lymphoid tissues and S1P abundance in lymph was shown to create a gradient across LECs, which induces transmigration of S1P_1_-expressing T cells into the lymphatic sinuses and egress into efferent lymph (93, 96): acting as a functional antagonist, FTY720 induces downregulation and degradation of S1P_1_ in T cells, thereby inhibiting S1P-mediated chemotaxis across the lymphatic sinuses (8). Similar to FTY720 treatment, adoptively transferred S1P_1_-deficient T cells were found to “log jam” around medullary and cortical sinuses and failed to egress into efferent lymph (8, 9, 23, 41). An analogous egress defect could also be evoked when the S1P gradient in LNs was experimentally destroyed, by inhibiting S1P lyase (77), or upon genetic deletion of Sphk1 and Sphk2 in LECs (41).

### Modulation of S1P_1_ for Fine-Tuning T Cell Transit Time through LNs

Similar to FTY720, high concentrations of S1P are capable of inducing S1P_1_ internalization in T cells (92, 97). Consequently, T cells in blood express low levels of S1P_1_ (95). Following entry into LNs *via* HEVs, T cells begin to upregulate S1P_1_ (95). Given that entry into LN sinuses, and subsequent egress from the LN, is S1P_1_ dependent, T cell transit time through the LN is in some manner dependent on S1P_1_-mediated resensitization to S1P in lymph. In addition to S1P-induced receptor internalization, the C-type lectin CD69 has also been reported to regulate S1P_1_ surface expression in T cells. CD69 is an early T cell activation marker and is upregulated in T cells by various inflammatory mediators, such as type I interferons (78, 93). CD69 has been shown to interact with S1P_1_, thereby inducing a receptor conformation similar to the ligand bound state, leading to S1P_1_ internalization and degradation (78, 79). CD69 expression by recently activated T cells therefore serves to inhibit the egress promoting function of S1P_1_ (24, 78, 79). However, activated T cells only transiently express CD69 (98). Accordingly, once activated T cells have undergone several rounds of division and have downregulated CD69, they start to re-express S1P_1_ and appear in circulation (8, 22). Akin to CD69 regulated surface expression of S1P_1_ on recently activated T cells, T cell receptor signaling (the first signal of T cell activation) has been reported to induce transcriptional downregulation of S1P_1_ (8). Transcriptional restoration of S1P_1_ is also likely to regulate T cell egress during an immune response.

### Role of CCR7

In addition to S1P_1_, CCR7 expression levels in T cells also impact the time T cells spend in LNs. Upon antigen recognition, activated T cells downregulate CCR7 (22). Fibroblastic reticular cells within the LN produce CCL21 and help generate a gradient where CCL21 levels are highest toward the LN center and decrease toward the peripheral medullary areas (25, 99). In addition to mediating intranodal positioning, migration, and motility (75), CCR7 also confers T cell retention within LNs (22). T cells devoid of CCR7 (CCR7^−/−^) egressed more rapidly than their wild-type (WT) counterparts, whereas transgenic T cells overexpressing CCR7 were retained in the LN for longer periods of time (22). Treatment with pertussis toxin restored egress competence of S1P_1_-deficient lymphocytes and in mixed bone marrow chimeras FTY720 treatment increased the number of CCR7^−/−^ T cells found in efferent lymph relative to their WT counterparts (22). Collectively, these findings suggest that CCR7 on T cells promotes their retention in LNs and that egress signals through S1P_1_ in part overcome CCR7-mediated retention (22). Interestingly, more CCR7^+/−^ than WT T cells entered sinuses, suggesting that the interplay between CCR7-mediated retention and S1P_1_-mediated egress occurs at the level of entry into sinuses (22). More recently, it has also been reported that C–X–C chemokine receptor 4 (CXCR4) on T cells synergizes with CCR7 to retain both naïve and activated T cells in LNs (80).

### Adhesion Molecules Involved in Egress across Sinuses

While it is well established that adhesion molecules and their integrin ligands play an important role in T cell entry into LNs through HEVs (100), not much is known about their role in T cell egress across lymphatic sinuses. A role for leukocyte function-associated antigen 1 (LFA-1) in delaying egress of T cells across lymphatic sinuses has recently been suggested. Following the probing of the surface of LN sinuses, CD4^+^ T cells devoid of LFA-1 had a greater tendency to egress across sinuses and spent less time in the LN than their WT counterparts (26). This distinction was lost in mice lacking the major LFA-1 ligand intercellular adhesion molecule 1 (ICAM-1) (26).

In addition to LFA-1, the common lymphatic endothelial and vascular endothelial receptor-1 (CLEVER-1), as well as the macrophage mannose receptor (MR) or its ligand L-selectin have been implicated in T cell migration across lymphatic sinuses: when performing adhesion assays on LN sections, antibody-mediated blockade of CLEVER-1 or MR reduced binding of lymphocytes to sinus endothelium (81, 82). However, the *in vivo* involvement of these receptors in LN egress has not been demonstrated thus far. On the other hand, a possible role for the integrin α9 subunit in lymphocyte egress from inflamed LNs has recently been reported (83). Integrin α9β1 is a well-described binding partner of the extracellular matrix component tenascin-C, and both α9 and tenascin-C reportedly are upregulated in medullary and cortical LN sinuses during inflammation. The study revealed that tenascin-C binding to LEC-expressed α9β1 induced S1P production in LECs, establishing a mechanistic link between α9 integrin expression and S1P_1_-mediated T cell egress. In fact, antibody-based blockade of α9 or tenascin-C deficiency resulted in impairment of T cell egress from inflamed LNs, reminiscent of treatment with FTY720 (83).

### Cellular Insights into Egress from Intravital Microscopy (IVM)

T cell egress from LNs has not only been studied at the population level but also at the single-cell level using IVM (see Box 1). Such studies have confirmed previous histology-based studies showing that T cell migration and egress occurs both at the level of the cortical and medullary sinuses (23, 101). T cells were observed entering sinuses at multiple locations, however, occasionally two or more T cells entered at specific entry “hot spots” (23, 101). In cortical sinuses without flow, T cells migrated at the same speed as those in the parenchyma and occasionally exited sinuses back into the LN parenchyma (23, 24). In larger cortical sinuses with flow, T cells were more rounded, shared fairly uniform velocities, and had a lower frequency of exit back into the parenchyma (23, 24). T cells in the macrophage-rich medullary sinuses appeared to become poorly mobile and occasionally exited the sinuses and returned to the T cell zone (23). Following migration of T cells through cortical and medullary sinuses, T cells were released into the subcapsular region near the efferent vessel and moved off rapidly with lymph flow (23). Overall, T cell transit time through the LN appears to be determined by random walk encounters with lymphatic sinuses (24). Only at the level of the sinus do S1P_1_-expressing T cells start to sense S1P in lymph, which triggers their exit into the lymphatic compartment (22–24).

## T Cell Entry and Migration within Afferent LVs

In comparison to T cell egress from LNs, little is known about T cell migration from peripheral tissue into afferent LVs. As already suggested by the dominance of CD4^+^ over CD8^+^ T cells in afferent lymph (43, 44, 62), CD4^+^ T cells migrate more efficiently through afferent LVs. Indeed, adoptive transfer studies (7), crawl-out experiments from murine skin explants (102), and studies in Kaede mice (31) uniformly demonstrate that CD4^+^ T cells more efficiently exit the tissue *via* afferent LVs. This is also reflected by emerging findings from many laboratories showing that under steady-state conditions most CD8^+^ T cells in peripheral organs form part of a slow-moving, skin-resident memory population [T_RM_; reviewed in Ref. (103, 104)]. Although recent studies indicate that a similar tissue-resident population also exists for CD4^+^ T cells (31), many CD4^+^ memory T cells seem to rapidly traffic through the dermis, forming part of a recirculating memory population (31, 102).

Although several molecules involved in T cell egress through afferent LVs have recently been identified, we still know fairly little about this process, particularly at the single-cell level. In fact, thus far only DC, but not T cell, migration through afferent LVs has been visualized using IVM (see Box 1). Interestingly, these findings have revealed that migration into and within afferent LVs occurs in a stepwise fashion: DCs enter LVs at the level of lymphatic capillaries and then crawl in a semi-directed manner within lymphatic capillaries (105–107). Only once they have reached contracting lymphatic collectors do cells switch from an active to passive mode of movement, i.e., they are passively carried away with the lymph flow toward the dLN. Similarly, neutrophils were recently found to actively crawl within dermal lymphatic capillaries (108). The reason why intralymphatic DCs and neutrophils only flow in lymphatic collectors is likely linked with the low flow conditions in lymphatic capillaries [reportedly ranging from 1 to 30 μm/s; (109, 110)], which are several orders of magnitude lower than blood flow in blood vascular capillaries (111) or peak lymph flow velocities measured in large contracting lymphatic collectors (112, 113). Although not demonstrated so far, it is therefore likely that T cell migration through lymphatic capillaries also involves an active, intraluminal crawling step (Figure 1A). In the following section, important molecules involved in T cell migration from the skin to the dLN will be discussed in greater detail (see also Table 2).

**Table 2:** **Molecules regulating T cell migration through afferent lymphatic vessels (LVs) into lymph nodes (LNs)**.

Molecule	Selected reference	Comment
CCR7	(7, 31, 114)	Adoptively transferred or endogenous CCR7^−/−^ T cells have reduced migration from peripheral tissues to dLNs
S1P_1/_S1P	(10, 12)	Treatment of adoptively transferred CD4^+^ T cells or recipient mice with FTY720 or S1P significantly reduces T cell migration to dLNs
CD44/mannose receptor (MR)	(115, 116)	T cell-expressed CD44 interacts with LEC-expressed MR during CD4^+^ and CD8^+^ T cell migration into afferent LVs
Common lymphatic endothelial and vascular endothelial receptor-1 (CLEVER-1)	(117)	CLEVER-1 blockade decreases CD4^+^ and CD8^+^ T cell migration from the skin to the dLN
LT and VCAM-1	(67)	Shown to mediate migration of nT_reg_ from skin to dLNs
Macrophage scavenger receptor 1	(118)	Regulates lymphocyte entry into the LN parenchyma
PLVAP (MECA-32)	(13)	Mediates lymphocyte entry across the subcapsular sinus into the LN parenchyma

### Chemotactic Exit and Retention Cues: CCR7, S1P_1_ and Others

Classical definitions outline that non-lymphoid tissue homing T_EM_ are devoid of CCR7 (119). However, in humans, CCR7 is expressed on the majority of T cells in blood, including those that express adhesion molecules required for homing to non-lymphoid tissue (120). Consistent with these findings, 40–50% of all skin-associated CD4^+^ T cells in humans (121) and mice (31) express CCR7. Several studies have identified CCR7 and its ligand CCL21, which is constitutive expressed by LVs (107, 122), as one of the most important drivers of T cell migration to dLNs: adoptive transfer experiments (7, 10) and experiments performed in Kaede mice (31) have shown that compared to WT T cells, significantly fewer (in the order of 10–20%) CCR7^−/−^ CD4^+^ or CD8^+^ T cells migrated from the skin to the dLN. Moreover, in a model of allergic airway inflammation, CCR7^−/−^ CD4^+^ T_EM_ cells accumulated in the lung and airways (114). Similarly, CD4^+^ T_EM_ have been shown to accumulate within the epithelial tissues of CCR7^−/−^ mice (123), and CCR7^−/−^ T_regs_ accumulated in inflamed skin (124). Although CCR7 appears to be crucial for T cell exit from homeostatic and acutely inflamed skin, its contribution to T cell exit from chronically inflamed skin appears to be more limited (11, 12). In the case of DCs, IVM studies have recently revealed that the CCR7/CCL21 axis mediates DC migration toward and into LVs (106, 122) and also impacts the directionality of DC crawling within lymphatic capillaries (107). By contrast, the exact contribution of CCR7 to T cell migration through afferent lymphatics has not been addressed so far.

Besides CCR7/CCL21, the second best described chemotactic pathway involved in T cell exit from skin is S1P_1_/S1P. As mentioned, LECs are considered the major contributor to S1P levels in lymph (41). Overexpression of S1P_1_ in CD8^+^ T cells prevented “settling” of T_RM_ in the intestine, kidney, salivary gland, and skin, suggesting S1P_1_ enhanced exit *via* afferent LVs (125). Similar to S1P_1_-overexpressing CD8^+^ T cells, CD69-deficient CD8^+^ T cells failed to persist in skin after HSV infection, and treatment with an S1P_1_ agonist restored their retention within the skin (126). Correspondingly, surface expression of CD69 and transcriptional loss of S1P_1_ is a hallmark for CD8^+^ T_RM_ (127–130).

In contrast to CD8^+^ T_RM_, tissue-resident CD4^+^ T cells have been less well characterized and studied. In a study using Kaede mice (see Box 1), Bromley and colleagues identified one population of CD4 memory T cells that remained in the skin and a second population, termed recirculating memory CD4^+^ T cells (T_RCM_), that migrated from the skin to the dLN (31). T_RCM_ expressed a novel cell surface phenotype (CCR7^int/+^, CD62L^int^, CD69^−^, CD103^+/−^, CCR4^+/−^, and E-selectin ligands^+^) and migrated in a CCR7-dependent manner (31). These cells displayed a trafficking behavior distinct from classical T_EM_ or T_CM_ cells in such that T_RCM_ migrated from skin to dLNs, and from circulation back into sites of unspecific cutaneous inflammation (31). The role of S1P in CD4^+^ T cell egress from skin has been addressed by two other recent studies (10, 12). Treatment of adoptively transferred T cells or of recipient mice with FTY720 or S1P significantly reduced T cell migration to the dLN (10, 12). Interestingly, acute inflammation was shown to increase S1P levels in the skin and also resulted in reduced migration of CD4^+^ T cells to the dLN (10). This suggests that acute inflammation might induce T cell retention in the tissue.

T cells that have migrated from the skin to the dLN display high expression of CCR7, CXCR4, and S1P_1_ (7, 10). In contrast to the involvement of CCR7 and S1P_1_, CXCR4 was reported to have no role in T cell migration from homeostatic (10) or inflamed skin to the dLN (11). By contrast, in a pancreatic islet transplantation model, CCR2, CCR5, and CXCR3 reportedly contributed to the migration of natural T_regs_ (nT_regs_) from the allograft to the dLN (66, 68). While LECs constitutively produce CXCL12, CCL21, and S1P (41, 131), they are also able to upregulate inflammatory chemokines under conditions of tissue inflammation (131, 132). This upregulation occurs in a stimulus-specific manner (131) and may serve to fine-tune leukocyte recruitment into LVs. Although not specifically studied so far, changes in the chemokine expression profile of LECs might also explain the reduced CCR7 and S1P dependence of T cell tissue exit observed from chronically but not from acutely inflamed skin (12). On the other hand, it has to be considered that most studies investigating T cell tissue exit have been performed using adoptively transferred T cells, which might not completely reflect the chemokine (or adhesion molecule) requirements of endogenous T cells.

### Adhesion Molecules Involved in Entry and Migration within Afferent LVs

#### MR and CLEVER-1

Few adhesion molecules have thus far been implicated in T cell exit from skin. The MR (82), which has been shown to mediate T cell binding to lymphatic sinuses in LNs (82), is also expressed on efferent and afferent LVs (133, 134). Interaction of MR with its T cell-expressed binding partner CD44 reportedly mediates CD4^+^ and CD8^+^ T cell exit from the skin (115, 116). Similarly, CLEVER-1 is expressed on both efferent and afferent LVs and has been shown to mediate T cell entry into afferent LVs (81, 117, 135). Blockade of CLEVER-1 markedly decreased CD4^+^ and CD8^+^ T cell migration from the skin to the dLN in both mice and rabbits (117).

#### VCAM-1, Selectins, and Their Ligands

A recent study suggested a role for LEC-expressed VCAM-1 in homeostatic migration of nT_reg_ but not of naïve CD4^+^ or CD8^+^ T cells from skin to the dLN (67). VCAM-1 is a known target of LTβR (136) and blockade of LTβR reduced nT_reg_ exit from the skin (67). Similarly, fewer nT_reg_ devoid of the LTβR ligand, LTα, exited from the skin (67). As with ICAM-1, VCAM-1 expression is induced on afferent LVs during inflammatory conditions (131, 132). Whether VCAM-1 might more broadly support T cell migration through afferent LVs in the context of tissue inflammation remains to be determined. With regard to the involvement of selectins, T cell migration from homeostatic skin to dLNs was found to occur normally in mice lacking the ligands for P-, E-, and L-selectins or upon adoptive transfer of CD62L^−/−^ T cells (10). However, it is noteworthy that P-selectin is also upregulated on afferent LVs during contact hypersensitivity-induced inflammation (131). This raises the question whether inflammation-induced selectins and their ligands might play a role in T cell exit under inflammatory conditions.

#### Insights into T Cell Entry into the LN from Afferent LVs

While several studies highlight the entry of T cells through HEVs or the migration of T cells within LNs (75, 137), few have focused on the entry of T cells into LNs from afferent LVs. Braun and colleagues investigated this entry pathway by performing time-lapse imaging in the popliteal LN following microinjection of T cells directly into the cannulated afferent LV (25). This study revealed that most naïve CD4^+^ T cells were passively transported in the SCS to peripheral medullary sinuses where they either directly transmigrated, or first crawled within the peripheral medullary sinuses before transmigrating into the LN parenchyma at the level of the medullary sinuses (25). As reported for T cell egress from the LN parenchyma into lymphatic sinuses (23, 101), several T cells occasionally crossed the sinus floor at specific transmigration “hot spots” (25). Interestingly, naïve CD4^+^ T cells entered across the medullary sinuses in a CCR7-independent manner, but subsequently preferentially migrated within the medulla toward the paracortical T cell zone by means of a CCR7-skewed random walk (25).

In contrast to T cells, injected DCs were able to directly transmigrate the SCS floor of the LN, allowing for a more direct access of the LN parenchyma (25). On the other hand, T cells injected after pre-injection of DCs now transmigrated the SCS floor on the afferent side of the LN and avidly migrated inward at sites of DC transmigration (25). These findings suggested that DCs induced local changes in the SCS floor during transmigration that facilitated direct entry of T cells into the LN parenchyma. Considering that afferent lymph typically contains both T cells and DCs that arrive simultaneously in the subcapsular space, it will be interesting to further explore LN entry from afferent LVs in an endogenous setup.

Other studies have suggested that T cells might enter the LN parenchyma directly through the SCS: as early as 4 h after adoptive transfer into the footpad of mice, T cells could be detected within the LN parenchyma in close proximity to the SCS (13, 118). Moreover, macrophage scavenger receptor 1, a molecule expressed on LECs of the SCS, but not on the medullary or cortical sinuses, was recently found to regulate lymphocyte entry into the LN parenchyma (118). Furthermore, the same group previously reported the involvement of plasmalemma vesicle-associated protein (PLVAP, also known as MECA-32) in lymphocyte entry across the SCS into the LN parenchyma (13). PLVAP is expressed by LECs in lymphatic sinuses where it forms diaphragms that overlay the entry to the FRC conduit system. This generates a sort of molecular sieve that restricts the access of soluble antigen into the conduit system and hence into the LN parenchyma. Interestingly, PLVAP also appeared to regulate T cell entry into the LN, supposedly by supporting transcellular diapedesis across the SCS (13).

## Purpose of T Cell Migration through Afferent LVs

As we gain more insight into T cell trafficking through LVs, our knowledge regarding the biological significance of this migratory process continues to grow. In the case of migration through efferent LVs, there is overwhelming evidence that this migratory step is crucial for immune surveillance: naïve T cells and T_CM_ constantly recirculate through blood, SLOs, and lymphatics in pursuit of antigen (1–3). Blocking this important migratory step, e.g., with FTY720, inhibits T cells recirculation and represents a powerful strategy for inducing immunosuppression, e.g., in the context of autoimmunity. On the other hand, recent data indicate that T cell trafficking through afferent LVs may not only occur to promote immune surveillance but may additionally have immune-dampening effects and serve to avoid overshooting T cell-mediated inflammatory responses. In the following section, these hypotheses shall be discussed in greater detail.

### Role of T Cell Circulation through Afferent LVs in Immune Surveillance

T cell recirculation through afferent LVs is thought to contribute to immune surveillance by constantly replenishing the T cell pool in peripheral tissues with new antigenic specificities. However, increasing evidence suggests that recirculating T cells do not provide complete protection of peripheral tissues, and that T_RM_ play a more important role in this process (104, 138). Although mainly studied for CD8^+^ T cells and in a limited number of infection models, T_RM_ (typically CD69^hi^, CD103^hi^, E-cadherin^hi^, ${\text{S1PR}}_{1}^{\text{lo}}$, and CCR7^lo^) have been shown to provide immediate protection against reinfection (104, 139). Current evidence suggests that T_RM_ differentiate from T_eff_, remain resident within the tissue for long periods of time (>1 year in mice) and predominate at sites of infection or inflammation (104, 140, 141). Although there is some evidence that T_RM_ proliferate locally, it is unknown whether T_RM_ are ever replaced by circulating T cells (139, 142, 143). The protective mechanisms of T_RM_ are not yet fully known, but evidence suggests that T_RM_ functionally delay pathogen spread and further act as an antigen-specific sensor that “sounds the alarm” for the recruitment of circulating T cells (104). The relative contribution of resident and circulating T cells in pathogen clearance remains unknown and might be highly context dependent, e.g., dependent on the type of infection and the specific requirement for CD4^+^ or CD8^+^ T cells for immune control (104, 139).

### Role of T_reg_ Tissue Exit in Controlling Immune Responses in dLNs

Previous studies have shown that the local ratio of T_regs_ to T_eff_ at inflamed sites is a critical determinant for the outcome of inflammation (144–146). In support of this notion, adoptively transferred CCR7^−/−^ T_regs_ that accumulated in the skin of mice controlled Th1-mediated inflammation more efficiently than WT T_regs_ (124). While these findings suggest that retention of T_regs_ within peripheral tissue promotes resolution of inflammation, large numbers of T_regs_ reportedly exit the skin during a cutaneous immune response in mice (30).

CD4^+^ T_regs_ control both priming and expansion of T_eff_ in SLOs and the activation of T_eff_ in the skin (147–150). Several islet allograft survival studies highlight T_reg_ migration to dLNs as a prerequisite for efficient downregulation of the ongoing allograft response (66–68, 151). Only T_regs_ within the skin, or having previously exited the skin *via* afferent LVs, reportedly displayed an activated phenotype (66). Upon adoptive transfer of egress-incompetent T_reg_ into the graft, graft survival was shorter than that for WT T_regs_ (66–68). Similarly, in a study using Kaede mice, T_regs_ that migrated from inflamed skin had an activated phenotype, inhibited immune responses more robustly than LN-resident T_regs_, and were able to recirculate back to the skin (30). These findings suggest that T_regs_ that have exited the skin *via* afferent LVs restrict LN immune responses (and consequently tissue inflammation) and recirculate back to inflamed tissue to help further control local immune responses.

### Role of Tissue Exit of Bystander T Cells in Resolving Local Inflammation

The extent of tissue inflammation often correlates with the number and composition of infiltrating T cells, which itself is dependent on T cell recruitment from blood, survival in the tissue, and, last but not least, on T cell exit through afferent LVs. Interestingly, two recent studies have shown that the ability of T cells to exit inflamed tissues has an impact on the degree of tissue inflammation. In mouse models of delayed-type hypersensitivity and TNF-driven Crohn’s-like ileitis, reduced exit of CCR7^−/−^ T cells from the site of inflammation translated into enhanced and prolonged inflammation (152, 153). Similarly, T cells overexpressing CCR7 had an enhanced capacity to exit from inflamed skin and accelerated resolution of inflammation (152). However, depending on the experimental setup, these experiments might have to be interpreted with caution because of the confounding influence of autoimmunity observed in CCR7^−/−^ mice, which might be due to other factors in addition to limited exit from peripheral tissues (76).

While recruitment into tissue is independent of the antigen specificity of T cells (154, 155), exit of T cells from inflamed tissues appears to be at least in part antigen dependent (152, 156). In a mouse model of delayed-type hypersensitivity, transgenic CD4^+^ Th1 cells, co-injected with DCs that were pulsed with cognate antigen, displayed reduced migration from inflamed skin to the dLN relative to polyclonal CD4^+^ Th1 cells (152). Similarly, a significantly reduced number of antigen-specific cytotoxic CD8^+^ T cells (Tc1), in comparison to antigen-unspecific Tc1 cells, migrated from the lung to the dLN in influenza-infected animals (156). These findings suggest that upon recognition of antigen, T cells have an impaired “tissue exit program” and are retained at the effector site, while antigen non-specific bystander T cells continue to exit *via* the afferent LVs in a CCR7-dependent manner (156). This mechanism is likely in place to reduce unnecessary tissue damage through bystander T cells.

## Conclusion and Outlook

In addition to cannulation studies, which have for more than six decades provided insights into the cellular composition of lymph, newer techniques such as adoptive transfer studies, LN egress studies or experiments performed in Kaede mice have considerably accelerated our recent gain of knowledge regarding the molecular mechanism of T cell trafficking through LVs. At the same time, IVM studies have provided further insight into the dynamics of these processes, by visualizing the single-cell behavior and anatomic location of T cell migration toward, across, and within LVs. While T cell egress from LNs into efferent LVs has been quite intensively studied, we still know comparably little about T cell migration into and within afferent LVs, or about the subsequent T cell entry step into the parenchyma of a dLN. In the future, it will be important to better characterize the distinct T cell subsets migrating through afferent LVs and the molecules involved in their trafficking. Moreover, the importance of tissue-resident vs. recirculating memory T cells will need to be addressed in more models. Given that CD4^+^ T cells constitute the main cell types recirculating through afferent LVs, this will be particularly relevant in the case of CD4^+^ T cell-dependent immunity. At the same time, it will also be important to carefully chose the right animal models when studying these processes: the fact that only few memory T cells are present in peripheral tissues of laboratory mice held under optimized hygienic conditions, and that these mice respond differently to immunologic challenges in comparison to mice housed under less hygienic environments (74), indicate that our preferred experimental setups might not represent a faithful model for studying the importance of recirculating vs. tissue-resident T cells in immune recall responses. On the other hand, we also still know very little about the potential function of recirculating T cells in dampening acute immune responses, possibly *via* tissue exit of T_regs_ or of T cells with irrelevant antigenic specificities. Recent observations that migration of DCs and neutrophils through afferent LVs involves active semi-directed migration within lymphatic capillaries suggest that the migratory process itself is more complex and might serve other purposes than the mere transport of cells to the dLN. Thus, in spite of recent advances regarding the molecular control of T cell traffic through LVs, we still know little about the biological relevance of these processes, particularly with regard to migration through afferent LVs. Ultimately, more insight into both the molecular mechanisms and the relevance are expected to contribute to identifying new targets for immunomodulatory therapies.

## Author Contributions

All the authors jointly wrote the manuscript and prepared the figures.

## Conflict of Interest Statement

The authors declare that the research was conducted in the absence of any commercial or financial relationships that could be construed as a potential conflict of interest. The reviewer TL and handling Editor declared their shared affiliation, and the handling Editor states that the process nevertheless met the standards of a fair and objective review.
